# A case of TAFRO syndrome after vaccination, successfully treated with cyclosporine

**DOI:** 10.1186/s12882-024-03630-x

**Published:** 2024-06-13

**Authors:** Yasuyuki Mimura, Katsuhito Kojima, Arisa Fujikawa, Shioko Okada, Akira Fujimori, Akihiro Kuma, Takahiro Kuragano

**Affiliations:** 1 Department of Cardiovascular and Renal Medicine, Hyogo Medical University, 1-1 Mukogawa-Cho, Nishinomiya, Hyogo 663-8501 Japan; 2 Department of Nephrology, Konan Medical Center, 1-5-16 Kamokogahara, Higashinada-Ku, Kobe, Hyogo 658-0064 Japan

**Keywords:** TAFRO syndrome, COVID-19, BNT162b2 mRNA, MIS-A (multisystem inflammatory syndrome in adults), SCLS (systemic capillary leak syndrome), ITP (idiopathic thrombocytopenic purpura)

## Abstract

**Background:**

TAFRO syndrome is a rare disorder that causes thrombocytopenia, generalized oedema, fever, organ enlargement, and renal impairment.

Few reports have suggested an association with vaccines, and few cases have undergone renal biopsy. TAFRO syndrome is often severe and fatal, and its cause is unknown. We report a case of TAFRO syndrome that occurred after vaccination with the coronavirus disease 2019 (COVID-19) vaccine.

**Case presentation:**

An 82-year-old woman received two doses of the BNT162b2 mRNA vaccine 3 weeks apart. Two weeks later, she was admitted to the hospital with oedema, accompanied with renal failure and thrombocytopenia. After close examination, she was diagnosed with TAFRO syndrome. She was treated with steroids, cyclosporine, and thrombopoietin receptor agonists.

The patient was discharged after several months in remission.

**Conclusions:**

Although an incident of TAFRO syndrome after COVID-19 vaccination has been previously reported, this is a rare case in which the patient went into remission and was discharged. A renal biopsy was also performed in this case, which was consistent with previous reports.

The favorable treatment course for TAFRO syndrome provides valuable insights.

## Background

TAFRO syndrome is a relatively rapidly progressive disease consisting of thrombocytopenia, profuse pleural fluid, ascites or generalized oedema, fever without apparent infection, increased megakaryocytes and mild hyperplasia of reticulin fibres in the bone marrow, renal dysfunction, and organomegaly [1].

After it was first proposed by Takai et al. [2] in 2010, it was compared with Castleman disease because of its similar histological findings. However, for a time it was an independent disease concept [3]. Later, its definition was proposed as an international standard, and TAFRO syndrome with histological Castleman-like findings was considered a subtype of Castleman's disease [4]. SARS-CoV-2 infection have caused a worldwide pandemic since 2019 and is a global concern. With the development of vaccines against COVID-19, such as BNT162b2 messenger ribonucleic acid (mRNA) vaccine (from Pfizer), SARS-CoV-2 infection and severity rates have been declining. The vaccine is highly effective, however, as with all other vaccines, there is a very low risk of developing serious side effects. Local injection site reactions, anaphylaxis, fever, and fatigue are common side effects, and cases of thrombosis and immune disorders have also been reported. In this manuscript, we report a case of TAFRO syndrome that progressed rapidly after vaccination with Pfizer's COVID-19 vaccine but improved to the point that the patient was discharged from hospital and was able to return to hospital as an outpatient after treatment. After reporting this case of TAFRO syndrome and its relation to vaccination, we review the relevant literature.

## Case presentation

The case analyzed herein was an 82-year-old female patient with a history of colonic diverticulosis and cerebral aneurysm (10 mm in size, no treatment). She denied any tobacco use or alcohol consumption, but she mentioned allergies to doxycycline and amoxicillin. No deterioration of renal or cardiac function had been reported in her previous medical examinations. Family history was unremarkable, too.

She had received the first and second doses of BNT162b2 vaccine for COVID-19 in June 20XX. On July 8, 20XX, she noticed that her neck had become thicker, so she visited a local cardiologist on July 15; furosemide 40 mg per day was prescribed for suspected renal and cardiac insufficiency.

On July 26, she consulted the local urologist, who prescribed azosemide 30 mg, although no improvement in her oedema was observed.

On July 29, she was referred to our hospital for further examination and treatment for suspected heart failure and she was admitted for further diagnostic testing and treatment because of marked oedema, worsening renal function with serum creatinine 1.98 mg/dL and a decreased platelet count of 4.0 × 10^4^ /μL.

### Physical symptoms on admission

On admission, her clinical findings were as follows: blood pressure, 112/92 mmHg; heart rate, 96/min; axillary temperature, 37.1 °C; body height, 153 cm; and body weight, 44.7 kg (body mass index 19.1 kg/m^2^).

She had severe oedema of the lower extremities and superficial lymph nodes in the cervical, axillary and inguinal regions could be palpated.

No arthralgias, neurologic findings, or skin lesions were noted. Furthermore, heart and lung examinations were normal.

### Blood/urine/pleural fluid examination: (Table 1)

**Table 1:** Laboratory findings on admission

Parameter	Value (reference range)
Biochemistry	
Total protein (g/dL)	5.5 (6.6–8.1)
Albumin (g/dL)	2.4 (4.1–5.1)
Total bilirubin (mg/dL)	0.6 (0.2–1.2)
Aspartate aminotransferase (U/L)	34 (13–30)
Alanine aminotransferase (U/L)	19 (10–42)
Lactate dehydrogenase (U/L)	158 (124–222)
Alkaline phosphatase (U/L)	531 (38-–113)
γ-Glutamyl transpeptidase (U/L)	23 (13–64)
Urea nitrogen (mg/dL)	15.8 (8–20)
Uric acid (mg/dL)	6.2 (3.7–7.0)
Creatinine (mg/dL)	1.98
eGFR (mL/min/1.73m^2^)	20.6
Na (mmol/L)	129 (138–145)
K (mmol/L)	4.7 (3.6–4.8)
Cl (mmol/L)	97 (101–108)
Ca (mg/dL)	7.2 (8.8–10.1)
P (mg/dL)	4.1 (2.7–4.6)
C-reactive protein (mg/dL)	11.06 (< 0.3)
Fe (μg/dL)	7 (64–187)
Ferritin (ng/mL)	293.5 (40–465)
UIBC (μg/dL)	121 (121–290)
Procalcitonin (ng/dL)	2.30 (< 0.05)
TSH (μIU/mL)	3.306 (0.5–5.0)
FreeT4 (ng/dL)	1.22 (0.9–1.7)
BNP (pg/mL)	320 (0.0–18.4)
Hematology	
White blood cell (/μL)	8.76 × 10^3^ (4.0–9.0 × 10^3^)
Seg (%)	78.0 (38.0–58.0)
Lym (%)	18.0 (26.0–47.0)
Mono (%)	4.0 (3.0–8.0)
Eos (%)	0.0 (2.0–7.0)
Red blood cell (μL)	292 × 10^4^ (380–500 × 10^4^)
Hemoglobin (g/dL)	8.2 (11.5–15.0)
MCV (fL)	89.4 (83.0–100.0)
Platelets (μL)	4.0 × 10^4^ (15.0–35.0 × 10^4^)
Coagulation	
PT (%)	108.3 (70.0–130.0)
APTT (second)	34.5 (24.0–34.0)
D-dimer (μg/mL)	10.3 (< 1.0)
Serology	
Immunoglobulin G (mg/dL)	1094 (870–1700)
Immunoglobulin A (mg/dL)	220 (110–410)
Immunoglobulin M (mg/dL)	36 (35–220)
Complement 3 (mg/dL)	79 (86.0–160.0)
Complement 4 (mg/dL)	19 (17–45)
CH50 (mg/dL)	46.6 (31.6–57.6)
ANA (times)	40 (speckled) (< 40)
Anti-dsDNA antibody (IU/mL)	17 (0.0–12.0)
Anti-SS-A antibody (U/mL)	57.8 (0.0–10.0)
Anti-SS-B antibody (U/mL)	3.7 (0.0–10.0)
MPO-ANCA (U/mL)	< 1.0
PR3-ANCA (U/mL)	< 1.0
Anti-GBM antibody (U/mL)	< 2.0
HIT antibody	Negative
PAIgG (ng/mL)	182 (< 46.0)
Anti-Helicobacter-antibody (U/mL)	36 (< 10.0)
ADAMTS13 activity (%)	18 (> 10.0)
Cryoglobulin	Negative
T-spot	Negative
Soluble interleukin-2 receptor (U/mL)	2230 (121–613)
Interleukin-6 (pg/mL)	23.1 (< 7.0)
Urinalysis	
Specific gravity	1.010
pH	5.5
Protein	2 +
Glucose	-
Occult blood	3 +
Bacteria	-
Red blood cell (/HPF)	40–49
Protein creatinine ratio (g/gCre)	0.18 (< 0.15)
Pleural fluid	
Color	Yellow and clear
PH	7.777
White blood cell (/μL)	0.162 × 10^3^
Red blood cell (/μL)	< 0.010 × 10^6^
Lactate dehydrogenase (U/L)	56
Proteins (g/dL)	2.4
Glucose (mg/dL)	121
Adenosine deaminase (U/L)	3.3
Cytology	No malignant
Cultivation	Negative

*UIBC* Unsaturated iron binding capacity, *PCT* Procalcitonin, *TSH* Thyroid stimulating hormone, *BNP* Brain natriuretic peptide, *Seg* Segmented neutrophil, *Lym* Lymphocytes, *Mono* Monocyte *Eos* Eosinophil, *MCV* Mean corpuscular volume, *PT* Prothrombin, *APTT* Activated partial thromboplastin time, *CH50* 50% hemolytic complement activity, *ANA* Anti-nuclear antibody, *ds-DNA* Double-stranded deoxyribonucleic acid, *Sm* Smith, *SS-A* Sjogren’s syndrome-A, *SS-B* Sjogren’s syndrome-B, *MPO-ANCA* Myeloperoxidase-anti-neutrophil cytoplasmic antibodies, *PR3-ANCA* Proteinase-3-anti-neutrophil cytoplasmic antibodies, *GBM* Glomerular basement membrane, *HIT* Heparin-induced thrombocytopenia, *ADAMTS13* A disintegrin-like and metalloproteinase with thrombospondin type 1 motifs 13, *PAIgG* Platelet-associated IgG, *T-spot* T-cell spot of tuberculosis assay, *IL-2* Interleukin-2, *IL-6* Interleukin-6

Blood test results on admission showed renal dysfunction, low platelets, and elevated inflammatory markers; an infection was initially on top of the differential diagnosis.

**Fig. 1:**
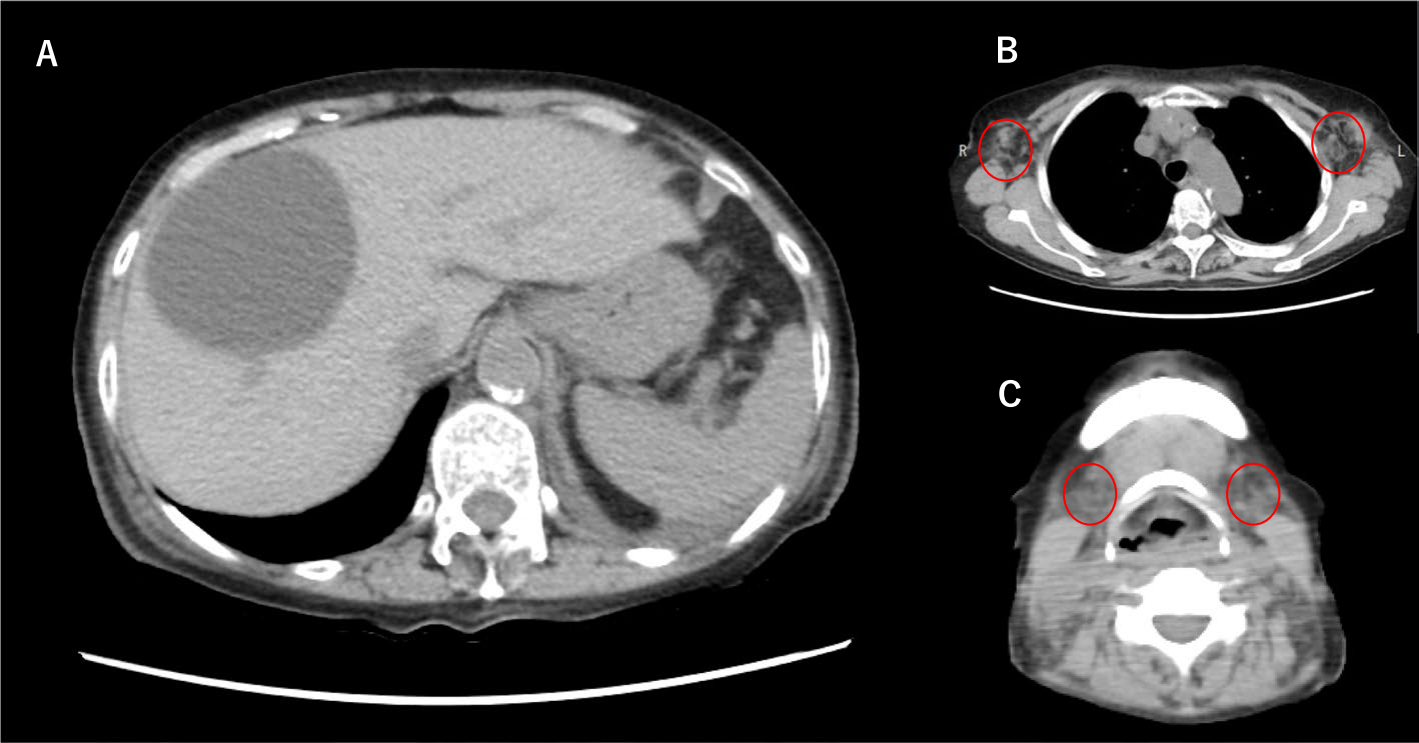
Computerized tomography imaging. **A** The liver is enlarged, and the spleen is mildly enlarged. **B**, **C** Scattered lymph node enlargement (< 1.5 cm) is noted at the neck, axillae and abdomen

**Fig. 2:**
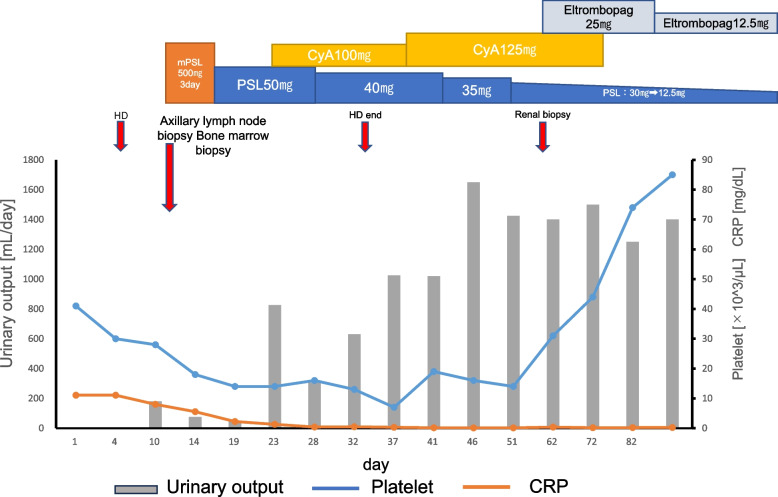
Clinical course of the patient. CRP, C-reactive protein; mPSL, methylpredonisolone; PSL, prednisolone; CyA: cyclosporine A; HD, hemodialysis

**Fig. 3:**
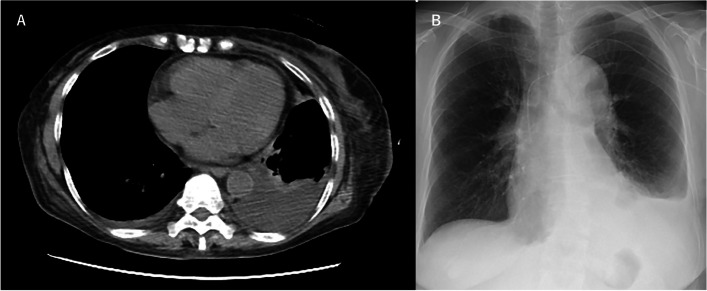
Chest X-ray and computed tomography imaging taken on the 6th day of hospitalization. **A**, **B** Pleural effusion increased on the left side compared to results at admission

**Fig. 4:**
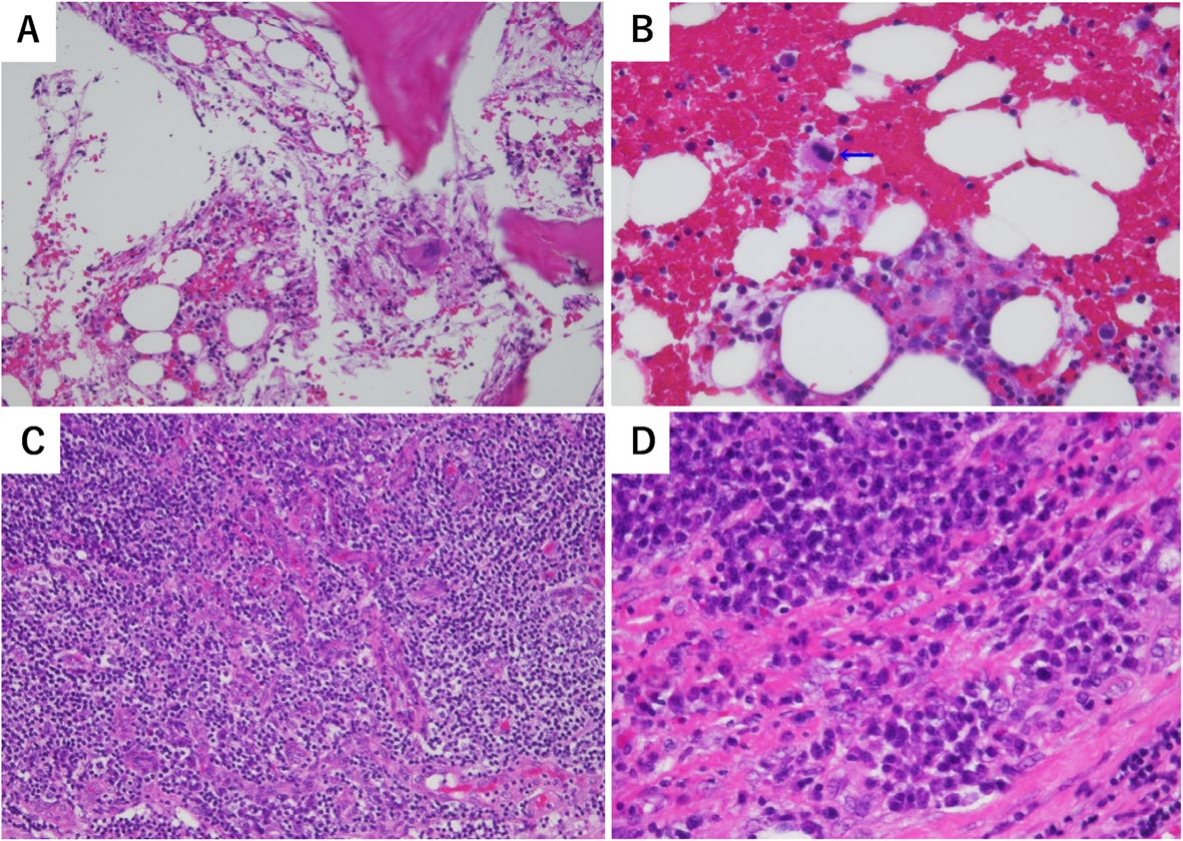
Pathological imaging. **A**, **B** Bone marrow biopsy: Mild fibrosis and megakaryocytosis are observed, without malignant findings or increase in blasts and plasma cells. (Periodic acid–Schiff staining, × 400). **C**, **D** Axillary lymph node biopsy: Resinous vascular growth and an increased number of plasma cells are observed. No malignant findings, such as cellular degeneration. (hematoxylin and eosin staining, × 100)

**Fig. 5:**
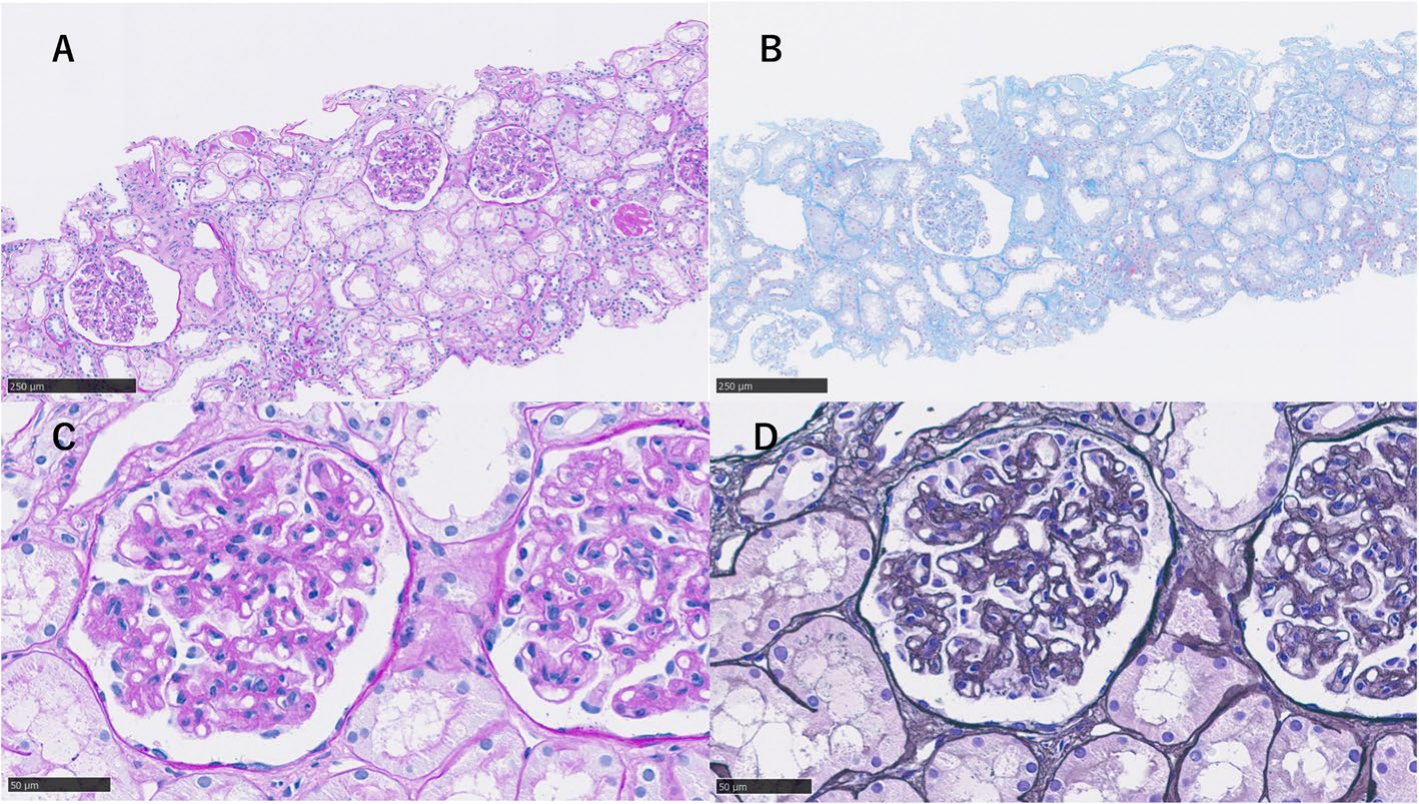
Renal biopsy imaging on light microscopy. **A**, **B** Periodic acid–Schiff staining and Masson trichrome staining, × 100: Total of 7 glomeruli, one obsolescence, semilunar formation ( −), Adhesions ( −). **C** Periodic acid–Schiff staining, × 400: Mesangium substratum is generally thickened. Thickening of the hoof wall and narrowing of the hoof cavity are observed. **D** Periotic acid methenamine silver staining, × 400: Doubling of the mooring hoof wall is observed. No nodule formation is observed.

However, since generalized oedema, cardiac enlargement and pleural effusion on chest radiograph were not consistent with the course of an infection, antibiotics were not started. A computerized tomography scan taken on admission showed enlarged bilateral cervical, axillary and intra-abdominal lymph nodes, mild hepatomegaly and splenomegaly (Fig. 1). The clinical course is shown in Fig. 2. Chest X-ray and computerized tomography images taken on 6th day of hospitalization revealed significantly increased pleural effusion (Fig. 3). In addition, the patient gained > 5 kg of body weight, her urine output decreased, and fluid control with diuretics was difficult, so a non-cuffed catheter was inserted through the right femoral vein, and hemodialysis was started. Diuretic-resistant oedema, worsening pleural effusion, and progressive thrombocytopenia were observed, and TAFRO syndrome was suspected at this point. Human herpesvirus 8 and peripheral blood smears were not tested in this case. On the 13th hospital day, biopsies of a left axillary lymph node and bone marrow were performed; no findings compatible with infection and malignancy were observed. As a result, a pulse therapy with 500 mg of methylprednisolone was administered from the 14th day for 3 days, followed by 50 mg of prednisolone daily. Mild fibrosis and megakaryocytosis were present in bone marrow biopsy (Fig. 4A-B). The lymph node biopsy revealed Castleman-like findings (Fig. 4C, D), and the patient was diagnosed with TAFRO syndrome according to the diagnostic criteria proposed in 2019 [1]. After starting prednisolone, although her urine output increased, this was not sufficient, and her platelet count was dependent on platelet transfusions. Considering the effect of these treatments insufficient, we started her on 100 mg of cyclosporine on the 23rd day. A pleural fluid examination was performed on the 33rd day to determine the pleural fluid component, which was a leaky pleural effusion. The dose of cyclosporine was increased to 125 mg on the 43rd day based on therapeutic drug monitoring. The prednisolone dose was reduced by 5 to 10 mg every week or two. The patient was weaned from dialysis on the 34th day of hospitalization because her urine output had stabilized, and her platelet count began to increase on the 51st day. Since the platelet count increased independently of transfusion, a renal biopsy was performed on the 58th day. The renal biopsy results revealed membranous proliferative glomerulonephritis (MPGN) findings consistent with TAFRO syndrome (Fig. 5).

**Fig. 6:**
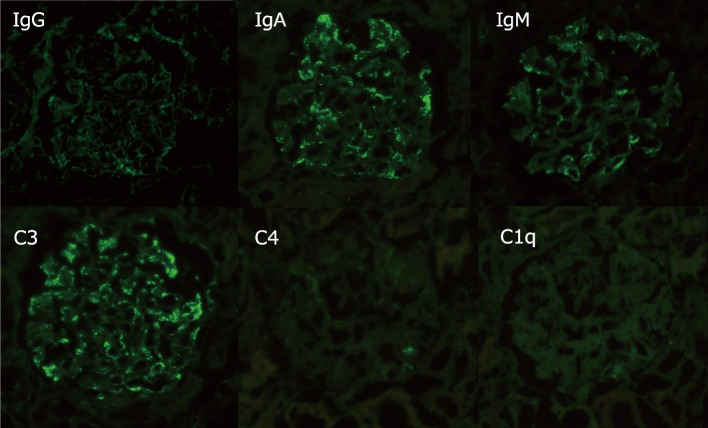
Immunofluorescence imaging of renal biopsy. Granular deposition of IgA and C3 on the capillary wall is observed. Other (IgG, IgM, and C1q) depositions were negative

Immunofluorescence staining was positive for immunoglobulin (Ig)A and complement 3 and negative for IgG, IgM, and Fibrinogen (Fig. 6).

**Fig. 7:**
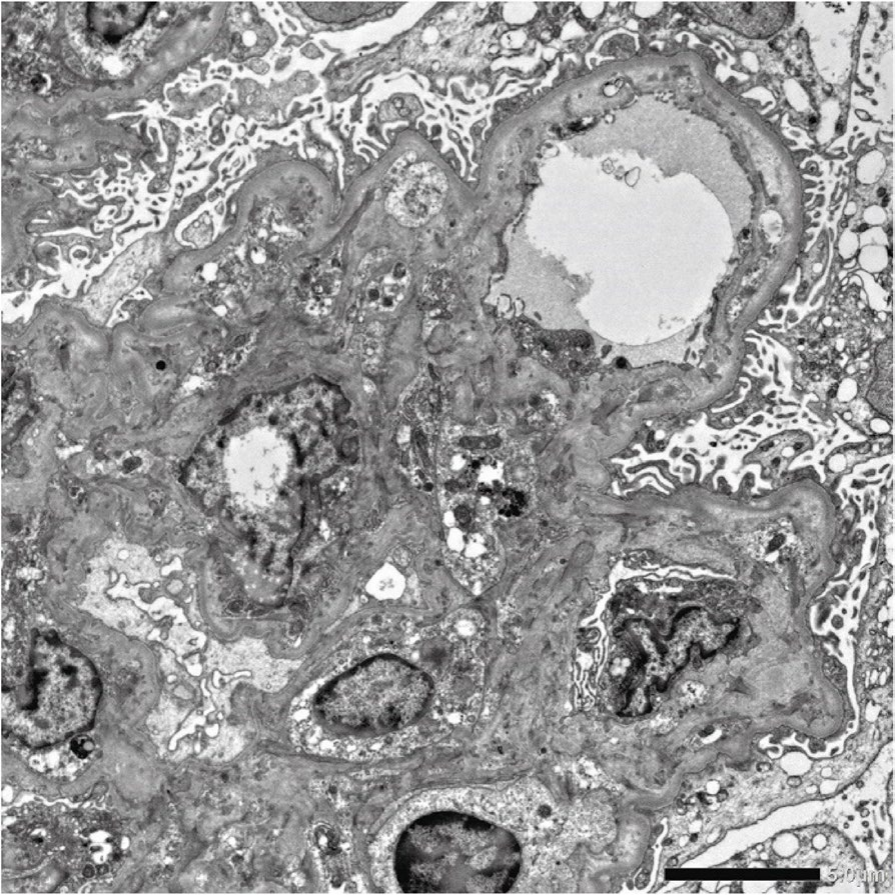
Electron microscopy imaging of renal biopsy. Electron microscopy: Edematous enlargement of the subendothelial space. Edematous changes in the mesangial area without deposits are noted

The electron micrograph revealed oedematous enlargement of the subendothelial space. The edematous changes in the mesangial area were observed, although no electron-dense deposits were detected (Fig. 7). After renal biopsy, eltrombopag 25 mg, a thrombopoietin receptor agonist, was administered to the patient, causing a sustained increase in platelet count, and the patient was discharged on the 108th day. At the time of discharge, the patient was taking 12.5 mg of prednisolone and 125 mg of cyclosporine.

## Discussion

**Fig. 8:**
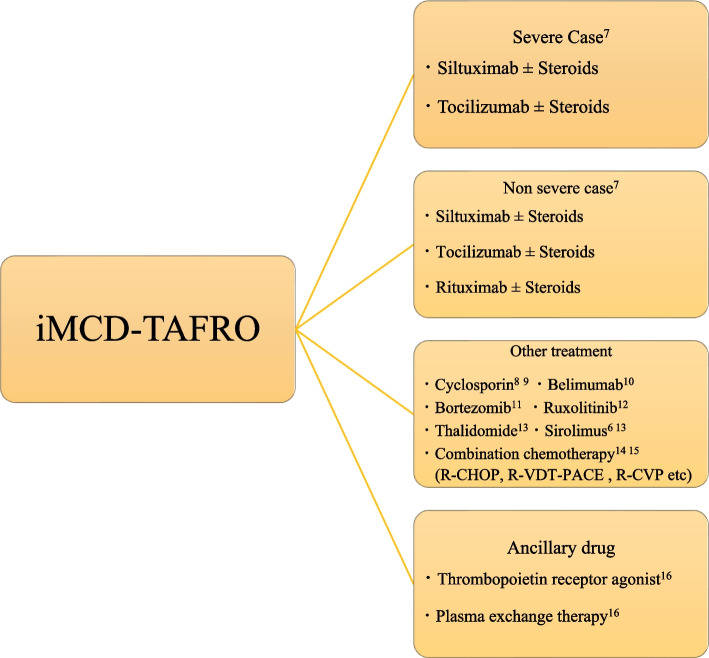
International standard treatment guidelines and previously reported treatments. R-CHOP, rituximab, cyclophosphamide, doxorubicin, vincristine, prednisone; R-VDT-PACE, rituximab, bortezomib, dexamethasone, thalidomide, cisplatin, doxorubicin, cyclophosphamide, etoposide; R-CVP, rituximab, cyclophosphamide, vincristine, prednisone

TAFRO syndrome has been increasingly reported since 2019, after diagnostic criteria from Masaki et al. were published [1]. Major criteria include oedema, thrombocytopenia, and inflammatory findings such as fever and elevated C-reactive level. Meanwhile, minor criteria include fibrosis of the bone marrow, mild organ enlargement, and progressive renal dysfunction. Although diagnostic criteria have been established, the pathogenesis of TAFRO syndrome is largely unknown. The disease concept demonstrates similarity with Castleman's syndrome, a systemic inflammatory disease caused by overproduction of the cytokine interleukin (IL)-6 because of the similar pathology. However, TAFRO has many different findings, including low platelets, oedema, and globulin counts [5]. Under current international criteria, any histologically similar finding to Castleman's disease is treated as a subtype of idiopathic multicentric Castleman's disease (iMCD), which includes this case [4]. Masaki et al. classified severity according to the degree of anasarca, thrombocytopenia, inflammation, and renal insufficiency, which in this case was grade 4 (severe) [1]. Age of > 60 years and D-dimer level > 18 μg/dL have been identified as potential prognostic factors in TAFRO syndrome, and only age is relevant in this case, classifying the present cases as an intermediate risk [6]. Although many studies have reported on this disease in Asian countries, including Japan, whether a racial factor exists is unclear. In Japan, reports of TAFRO syndrome in the country are scattered, and the prevalence of the disease concept may be a reason for the high number of TAFRO syndrome diagnoses. Treatment guidelines for iMCD recommend anti-IL-6 therapy and/or rituximab in addition to steroids, although no treatment strategy has been established specifically for iMCD-TAFRO [7]. The international standard treatment guidelines and previously reported treatments are briefly summarized in the Fig. 8 [7–16]. In this case, thrombocytopenia was the most significant clinical problem. Previous reports have demonstrated the efficacy of cyclosporine for thrombocytopenia in TAFRO syndrome, suggesting that inhibition of IL-2 may improve thrombocytopenia [8, 9]. Although IL-6 levels were high in this patient, cyclosporine was preferred because IL-6 levels have also been reported to be out of proportion to therapeutic efficacy [17].

Renal biopsy is rarely performed in patients with TAFRO owing to low platelets and other factors, although in many cases, thrombotic microangiopathy (TMA) and MPGN are detected. The present patient also presented with MPGN findings in light microscopy (Fig. 5), although since the chronic lesions of TMA can resemble MPGN and the renal biopsy was performed when platelets had recovered, TMA pathology may be involved [18–23]. Mizuno et al. [24] have described small arterial lesions with an adult onset in seven cases of TAFRO syndrome. Vacuolization of arteriolar myocytes was detected in all seven patients, and arteriolar hyalinosis was noted in four of them. However, in our case, no small arterial lesions were detected. IgA and complement 3 were positive on immunostaining, and we suspected a complication of IgA deposition. Immunofluorescent staining also demonstrated granular deposition of IgA on the capillary wall, which excluded IgA nephropathy (Fig. 6). Electron microscopy revealed expansion of the subendothelial space, and no immune complex deposition was observed in glomeruli (Fig. 7). These findings were reasonably considered a finding of TMA. We listed kidney histopathologic findings of previous reported cases of TAFRO syndrome in Table 2 [9, 19–22, 25–33]. All cases, including ours, demonstrated findings of a marked glomerular endothelial cell injury.

**Table 2:** List of renal histopathologic findings in previous reported recent cases of TAFRO syndrome

Author, year	Age	Sex	Country	Clinical symptoms	SCr (mg/dL)	UPCR (g/g･Cr)	Light microscopy	Immunofluorescence	Electron microscopy
Ozeki T, et al. [19] (2018)	51	Female	Japan	Abdominal distension Oedema	1.03	0.52	MPGN	All nagative	EDD ( −), endothelial cell swelling
Furuto Y, et al. [25] (2018)	55	Female	Japan	Dyspnea	2.10	0.54	MPGN	All nagative	EDD (-), Partial duplication of the basement membrane and mesangial interposition
Noda-Narita S, et al. [21] (2018)	79	Female	Japan	Fever Edema	1.85	2.65	Global duplication of basement membranes and mesangiolysis	All nagative	EDD (-), endothelial cell swelling
Mizuno H, et al. [20] (2018)	84	Male	Japan	Fever Oedema	2.31	0.3	MPGN, endothelial cell swelling and vacuolization	All nagative	EDD (-), endothelial cell swelling
Nakamori A, et al. [22] (2018)	54	Female	Japan	Fever Oedema	1.11	3.2	MGA, endothelial swelling	All nagative	EDD (-), endothelial cell swelling
Leurs A, et al. [26] (2019)	28	Female	France	Fever Oedema	1.19	2	MPGN	IgM( +) and C1q( +) on capillary loop	EDD ( +); intra-membranous focal deposit
Saito H, et al. [27] (2019)	45	Female	Japan	Abdominal pain Fever	0.64	0.2	MPGN	All nagative	EDD (-), endothelial cell swelling and widening of the subendothelial space
Nagayama Y, et al. [28] (2019)	48	Female	Japan	Oedema	1.32	1.57	MPGN	All nagative	EDD (-), edematous enlargement of the subendothelial space
Simeni Njonnou SR, et al. [29] (2020)	42	Female	Belgium	Abdominal pain	1.96	Unknown	MPGN	IgM( +) and C3( +) on capillary loop	Unknown
Zhou Q, et al., case1 [30], (2020)	30	Female	China	Oedema Vaginal bleeding	7.06	Unknown	Local aggravated endothelial cell proliferation, The basement membrane thickened heterogeneously, Onion skin appearance	IgM( +) on mesangial area and capillary loop	EDD (-), endothelial cell swelling and widening of the subendothelial space
Zhou Q, et al., case2 [30], (2020)	42	Male	China	Epigastric pain	3.11	Unknown	MPGN	Full house pattern (capillary loop)	EDD ( +); intramembranous, subendothelial, and partially subepithelial deposits
Shimada K, et al., case1 [31], (2021)	30's	Male	Japan	Fatigue	1.92	1.81	MPGN, Focal mesangiolysis and ballooning of glomerular capillary, loops Interlobular artery thrombus	Unknown	Unknown
Shimada K, et al., case2 [31], (2021)	50's	Male	Japan	Fever	1.20	1.31	Endothelial cell swelling and focal mesangiolysis	Unknown	EDD (-), endothelial cell swelling
Shimada K, et al., case3 [31], (2021)	50's	Male	Japan	Cough Fever	1.10	0.37	MPGN, focal mesangiolysis and ballooning of glomerular capillary loops	Unknown	EDD (-), edematous enlargement of the subendothelial space
Iwasaki T, et al. [9] (2022)	61	Female	Japan	Abdominal pain Abdominal distension	1.07	0.78	MPGN	IgM( +) on mesangial area	EDD (-), endothelial cell swelling, Edematous enlargement of the subendothelial space
Nakayama Y, et al. [32] (2023)	16	Female	Japan	Fever Oedema	4.40	2.38	MPGN, lumen narrowing of the interlobular arteries	All nagative	EDD (-), endothelial cell enlargement with subendothelial edema
Sato H, et al. [33] (2023)	51	Male	Japan	Fever	13.26	Unknown	MPGN	C3( +) and C1q( +) on capillary loop	EDD (-), endothelial cell swelling with expansion of the subendothelial space

*S-Cr* Serum creatinine, *UPCR* Urinary protein creatinine ratio, *MGA* Minor glomerular abnormality

As mentioned above, although some findings are different between idiopathic multicentric Castleman’s disease (not otherwise specified) and TAFRO syndrome, cytokines play an important role in both subtypes, as TAFRO syndrome is often associated with high IL-6 and vascular endothelial growth factor (VEGF) levels [17]. Based on previous reports, the high probability of high procalcitonin and high levels of presepsin suggests that TAFRO syndrome is likely secondary to a prior infection [34]. In most cases, the disease is preceded by an event that triggers an immune response, such as an infection, and genetic influences are unlikely. The high procalcitonin levels in this case were also considered secondary to a prior infection. However, no bacterial or viral infection was observed on systemic examination, and we speculated that the COVID-19 vaccine, which was administered before the onset of the disease, was the cause of the disease.

Although TAFRO syndrome caused by the BNT162b2 mRNA (Pfizer) vaccine has been previously reported [35], this is a rare case in which a patient was discharged from hospital in a condition that allowed outpatient hospitalization [36, 37].

Many previous cases of vaccine-induced immunological diseases have been attributed to the cytokine-boosting effect of adjuvant-containing vaccines. Adjuvants are administered with vaccine antigens to enhance antigen-specific immune responses in localized areas, such as the regional lymph nodes. Because adjuvants induce an inflammatory response in the host, higher doses and molecular weights not only result in more efficient transfer to the regional lymph nodes and a higher antigenic immune response but also increase the likelihood of a cytokine storm [38]. The BNT162b2 mRNA used in this case was an mRNA vaccine that did not contain an adjuvant. However, lipid nanoparticles, a drug delivery system that prevents mRNA degradation and efficiently delivers mRNA to target cells, contain lipids and polymers, and many of them exhibit adjuvant effects [39]. Therefore, even mRNA vaccines may cause cytokine overproduction, and the fact that two doses were administered within a 3-week period may have contributed to this patient’s clinical condition. Myocarditis occurring after vaccination has been reported, and myocarditis-induced heart failure was also considered in this case. However, heart function was normal on echocardiography, and the electrocardiogram did not indicate myocarditis, so heart failure including myocarditis was ruled out.

Adult multisystemic inflammatory syndrome (MIS-A), systemic capillary leak syndrome (SCLS), and idiopathic thrombocytopenic purpura (ITP) have been reported in association with COVID-19 vaccines [40–42]. MIS-A may cause multiorgan damage because of cytokine-induced organ inflammation, SCLS causes systemic oedema due to vascular endothelial cell damage, and ITP causes platelet depression due to the production of antiplatelet antibodies. IL-6, VEGF, and platelet-associated IgG are often elevated in TAFRO syndrome, as in this case, suggesting the possibility of a combined MIS-A, SCLS, and ITP lesion. When unexplained oedema and thrombocytopenia are observed after vaccination, TAFRO should be included in the differential diagnosis. We hope our case report will elucidate the pathogenesis of TAFRO syndrome.

## Conclusions

Although the patient developed TAFRO syndrome after COVID-19 vaccination, which is often severe, she recovered to the point where she was able to go to an outpatient clinic after administration of steroids and cyclosporine. If a similar condition develops after vaccination, TAFRO syndrome should be considered in the differential diagnosis.

## Data Availability

All relevant data are within the manuscript.

## Abbreviations

mRNA: Messenger ribonucleic acid
MPGN: Membranous proliferative glomerulonephritis
IL: Interleukin
iMCD: Idiopathic multicentric Castleman's disease
TMA: Thrombotic microangiopathy
Ig: immunoglobulin
VEGF: Vascular endothelial growth factor
MIS-A: Adult multisystemic inflammatory syndrome
SCLS: Systemic capillary leak syndrome
ITP: Idiopathic thrombocytopenic purpura
